# Dietary patterns and all-cause and cardiovascular disease, and cancer mortality in Korean adults

**DOI:** 10.1186/s12937-024-01064-7

**Published:** 2025-03-03

**Authors:** Soomin Lee, Jung Eun Lee, Minji Kang

**Affiliations:** 1 Department of Food and Nutrition, Seoul National University, Seoul, Republic of Korea; 2 Research Institute of Human Ecology, Seoul National University, Seoul, Republic of Korea; 3 Department of Food and Nutrition, Duksung Women’s University, Seoul, Republic of Korea

**Keywords:** Dietary patterns, Mortality, Korea national health and nutrition examination survey

## Abstract

**Background:**

Examining dietary patterns in relation to mortality offers a more comprehensive view of food and nutrient intake. However, to our knowledge, the association of the Korean population’s dietary patterns with mortality remains scarce and unclear. We aim to investigate the association between dietary patterns and all-cause, cardiovascular disease (CVD), and cancer mortality in the Korean population.

**Methods:**

This prospective cohort study included 18,019 men and 26,604 women aged ≥ 19 years who participated in the Korea National Health and Nutrition Examination Surveys 2007–2015. Dietary data were collected from a 24-hour dietary recall. Mortality from all causes, CVD, and cancer were ascertained from linkage to Statistics Korea. We identified dietary patterns through factor analysis. Cox proportional hazard models were used to estimate the hazard ratios (HRs) and 95% confidence intervals (CIs) of the all-cause, CVD, and cancer mortality according to diet pattern scores.

**Results:**

During a mean follow-up of 8.19 person-years, 2,622 deaths were identified, with 595 due to CVD and 827 due to cancer. Factor analysis based on 22 food groups, three dietary patterns were obtained: (1) “animal food and condiment pattern,” (2) “seafood and vegetable pattern,” and (3) “dairy products and processed meat pattern.” After multivariable adjustment, the “seafood and vegetable pattern” score was associated with a lower risk of all-cause and CVD mortality. HRs (95% CIs) for all-cause mortality for the second to the highest quintile of “seafood and vegetable pattern”, compared with the first quintile were 0.86 (0.75–0.99), 0.75 (0.65–0.87), 0.81 (0.69–0.94), and 0.87 (0.73–1.05), respectively (P for trend = 0.191). For CVD mortality, the HRs were 0.82 (0.62–1.07), 0.80 (0.58–1.08), 0.70 (0.50–0.99), and 0.63 (0.42–0.96), respectively (P for trend = 0.027). No statistically significant associations were found in “animal food and condiment pattern” or “dairy products and processed meat pattern” for all-cause, CVD, and cancer mortality.

**Conclusions:**

We observed that the “seafood and vegetable” dietary pattern decreased the risk of all-cause and CVD mortality in Korean adults.

## Introduction

Diet-related chronic diseases, such as cardiovascular disease (CVD) and cancer, are the primary causes of death worldwide [1]. According to the Global Burden of Disease Study, CVD was the primary cause of death related to diet worldwide in 2017, followed by cancer [2]. Cancer has surpassed CVD as the leading cause in most very high-Human Development Index countries [1]. Stroke remained the second and third-leading cause of death in 2019. The impact of stroke will persistently increase, disproportionately affecting countries with lower and middle incomes [3].

Traditionally, research on the association between diet and disease has focused on examining individual nutrients or specific foods. Over time, however, this focus has shifted, leading to the development of dietary pattern analysis, a complementary approach that offers an alternative approach that analyzes the consumption of several foods or food groups comprehensively [4]. Many prior studies have investigated the relationship between dietary patterns and mortality, as well as various diseases, including cancer, CVD, and type 2 diabetes [5–7].

In the UK biobank cohort, cumulative dietary risk factors were associated with higher risk for all-cause, CVD, and cancer mortality [8]. Another study using the UK Biobank cohort, a dietary pattern characterized by high intakes of chocolate and confectionery, butter and low-fiber bread, and low intakes of fresh fruit and vegetables and wholegrain foods, was positively associated with higher all-cause mortality [6]. In the US, the Third National Health and Nutrition Examination Survey (NHANES III) examined the association between dietary patterns and both overall mortality and cancer mortality. Adherence to the ‘western’ and the ‘traditional’ dietary pattern was strongly associated with an increased risk of total mortality and cancer mortality, while the ‘prudent’ dietary pattern was associated with a decreased risk of total mortality [7]. Nurses’ Health Study (NHS) showed similar results with NHANES. In the NHS, women with higher prudent pattern scores showed a reduced risk of cardiovascular and all-cause mortality. Conversely, women with higher scores on Western dietary pattern faced an elevated risk of CVD, cancer, and all-cause mortality [9].

In addition, a study of Asians, specifically the Japan Public Health Center-based Prospective Study (JPHC) [10, 11], also showed similar results with NHANES and NHS cohorts. The prudent dietary pattern, characterized by a high intake of vegetables, fruit, soy products, potatoes, seaweed, mushrooms, and fish, exhibited a significant association with a decreased risk of all-cause and CVD mortality. On the other hand, a Westernized dietary pattern consisting of meat, processed meat, bread, and dairy products was inversely associated with the risk of all-cause, cancer, and CVD mortality [10]. Another study [11], utilizing the JPHC study, analyzed the association between traditional Japanese dietary pattern and mortality risk. The Japanese diet, described as a high intake of rice, miso soup, seaweeds, pickles, green and yellow vegetables, fish, and green tea, and a low intake of beef and pork, assessed by the 8-Item Japanese Diet Index (JDI8), was linked to a decreased risk of all-cause and CVD mortality. Furthermore, findings from the Singapore Chinese Health Study highlighted the impact of specific dietary patterns on mortality: The VFS pattern (vegetable, fruit-, and soy-rich) demonstrated an inverse association with all-cause mortality and each cause-specific category (CVD, cancer, and respiratory) of mortality. Conversely, the DSM (dim sum- and meat-rich) dietary pattern showed a positive association with CVD mortality in the overall population [12].

To our knowledge, while the association between dietary patterns and mortality has been investigated globally, no studies have specifically focused on the Korean population. Given the distinct dietary habits in Korea compared to Western countries, applying findings from global studies may be challenging. Therefore, we aimed to examine the association of dietary patterns with all-cause, CVD, and cancer mortality in a large, nationally representative cohort study of Korean men and women.

## Methods

### Study population

This study utilized data from the Korea National Health and Nutrition Examination Survey (KNHANES). KNHANES, conducted by the Korea Centers for Disease Control and Prevention (KCDC), is a nationally representative survey that has been evaluating the health and nutritional status of the Korean population since 1998 [13]. This survey recruits approximately 10,000 individuals as a representative sample each year, and consists of health interview survey, health examination survey, and nutrition survey. The details of KNHANES have been described previously [13, 14].

Among 73,353 participants enrolled in the KNHANES 2007–2015, aged 19 years or older, we excluded individuals who did not consent to link their data to the Cause of Death Statistics, leading to missing information on mortality (*n* = 21,778). From the remaining 51,575 individuals who gave assent to the linkage, those with missing information on energy intake (*n* = 6,122) and individuals reporting implausible energy intake (> 3 standard deviations from the natural logarithm transformed mean) (*n* = 361) were excluded. Additionally, to minimize the probability of reverse causation, participants who died within 2 years of follow-up were excluded (*n* = 469). After these exclusions, a total of 44,623 (18,019 men and 26,604 women) were included in the current study.

The KNHANES protocol received approval from the Korea Centers for Disease Control and Prevention, and informed written consent was obtained fom all participants. This study was waived by the Institutional Review Boards of Seoul National University (IRB No.E2303/002–002).

### Calculation of dietary pattern score

We categorized food groups based on dietary intake data obtained through a single 24-hour dietary recall. Trained dietitians guided participants in recalling and reporting all foods and beverages consumed the day prior. The collected information encompassed food names, types of ingredients, and the amount of food intake per meal. Prior to conducting interviews, survey staff underwent comprehensive training [13], including intensive instruction and practical teaching for interviews. Additionally, retraining sessions were conducted to reinforce the appropriate protocols and techniques.

The daily energy and nutrient intake were calculated utilizing data from the Korean Food Composition Database, provided by the Rural Development Administration [14], ensuring confirmed validity and reliability. Nutrition and health examinations were conducted throughout the week, covering both weekdays and weekends. Until before 2013, health interviews and nutrition surveys were conducted through face-to-face interviews using paper-based methods. Afterward, the survey method transitioned to computer-assisted personal interviewing [15].

Dietary patterns were determined through factor analysis (PCA; principal component analysis) using daily consumption data from 22 food groups, adapted from KNHANES 24-hour dietary recall. The data, initially categorized into 19 food groups (grains, potatoes, sugars, starches, seeds and nuts, vegetables, mushrooms, fruits, seaweed, beverages and liquor, seasonings, oils(plants), etc. (plant), meat, eggs, fish and shellfish, dairy products, fats(animal), and etc.) based on the KNHANES food code system, underwent further subdivision. Grains were differentiated into whole grain and refined grain, while beverages and liquor were further classified into alcoholic beverages and non-alcoholic beverages. Meats were categorized into red meat, processed meat, and poultry meat. Subsequently, the categories, etc. (plant) and etc. (animal) were consolidated into a single category, labeled as, etc.

As 10 factors met the criteria for eigenvalues greater than one, we considered eigenvalues, the scree test, and interpretations of the factors based on the results from the factor analysis. Throughout these considerations, three dietary patterns emerged as the most meaningful representation of distinctive dietary patterns in the study population. Factor loadings with an absolute value ≥ 0.25 were used to identify variables that significantly contributed to each dietary pattern. This threshold was chosen based on previous studies [16, 17] and to better capture the characteristics of each pattern. Using this approach, we applied orthogonal rotation (Varimax) to simplify interpretation while retaining the most relevant variables for each factor. Based on the identified variables, the three major dietary patterns were named as follows: (1) “animal food and condiment pattern,” (2) “seafood and vegetable pattern,” and (3) “dairy products and processed meat pattern.”

Factor scores for each dietary pattern were calculated by summing the intakes of food groups, which were weighted based on their factor loadings. The factor loading indicates the extent to which a food group is correlated with the factor, and a negative factor loading implies an inverse correlation with the factor. Individuals with high dietary pattern scores had greater compliance with the following pattern compared to individuals with lower scores.

### Assessment of covariates

Sociodemographic factors, including age, sex, education level, income level, and marital status, as well as smoking status, alcohol consumption, and vigorous physical activity, along with medical history and dietary supplement usage, were acquired through health interviews and self-reported questionnaires. Participants provided details on their smoking status (never, past, or current smoker), average daily cigarette consumption among current smokers, and the duration of smoking in years. Additionally, participants disclosed their drinking status (never, past, or current drinker) for various types of alcohol, specifying frequency and quantity in glasses. Pack-years were calculated by dividing the number of cigarettes smoked per day by 20 and then multiplying this by the number of years the person has smoked. The overall ethanol intake was determined by summing up the product of frequency and quantity, while considering the standard ethanol content per glass. The KNHANES physical activity questionnaire was developed based on the Korean version of the International Physical Activity Questionnaire [18] between 2005 and 2014 and the Global Physical Activity Questionnaire [19] since 2014 [15]. The variable about vigorous physical activity is dichotomy. Individuals who reported engaging in vigorous physical activity for more than one day per week or indicated “yes” to performing vigorous physical activity were classified as “yes” for the variable. During the health examination, anthropometric measurements were conducted by well-trained medical personnel. Height was measured to the nearest 0.1 cm and weight to the nearest 0.1 kg, with participants wearing light clothing and no shoes. Body mass index (BMI) was calculated dividing weight (kg) by the square of the height (m^2^).

Nine people with pack-years of 3907.2 were considered outliers. The pack-years value of 3907.2 was replaced with the value corresponding to the top 1%, which was 225. This approach was undertaken to ensure the robustness of the data and to reflect a more accurate representation of the pack-years distribution [20]. Assitionally, we conducted the analysis without replacing the pack years value of 3907.2 with 225, and the results remained consistent.

### Ascertainment of mortality

We identified all-cause (2,622 deaths), cardiovascular disease (595 deaths), and cancer mortality (827 deaths) during an average follow-up of 8.19 person-years. The underlying causes of death were categorized according to the following International Statistical Classification of Diseases and Related Health Problems, Tenth Revision codes (ICD-10) [21] or Korean Standard Classification of Disease version 7 (KCD-7) codes [22]: disease of the circulatory system (I00-I99), and neoplasms (C00-D48).

### Statistical analysis

Participant characteristics are presented on quintiles of three major dietary pattern scores. Continuous variables were indicated as mean ± SE, and categorical variables were shown as either number (N) or percent (%). Cox proportional hazard regression analysis was used to compute hazard ratios and 95% confidence intervals (CIs) of all-cause, CVD, and cancer mortality in relation to quintiles of the dietary pattern score. The person-time of follow-up for each participant was calculated from the enrollment date until the date of death or the end of follow-up (December 31, 2019), whichever occurred first. Model 1 was stratified by age at baseline (19 to < 60 years and 60 + years) and adjusted for age (continuous, years) and sex. Model 2 was further adjusted for baseline year (2007 to 2015), total energy intake (continuous, kcal/day), BMI (10 to < 18.5 kg/m^2^, 18.5 to 23 kg/m^2^, 23 to < 25 kg/m^2^, 25 to < 30 kg/m^2^, ≥ 30 kg/m^2^), smoking status (never, past < 15 pack-years [py], past ≥ 15 py, current < 18.2 py, current ≥ 18.2 py for men; never, past < 1.05 py, past ≥ 1.05 py, current < 5.5 py, current ≥ 5.5 py for women), current alcochol intake (none, current < 3 drinks/week, 3 ≤ current < 7 drinks/week, current ≥ 7 drinks/week), marital status (married, not married), education (elementary school or below, middle school, high school or above), household income (low, low middle, upper middle, high), vigorous physical activity (yes, no), history of cancer, CVD, or diabetes (yes, no), and the use of supplements (yes, no). The median pack-years value within each group was used as the cutoff point within past smokers or current smokers.

Furthermore, we conducted subgroup analyses of all-cause mortality by sex (men and women), age at baseline (aged 19–59 and 60 + years), BMI (< 25, 25+), smoking status (nonsmoker, current smoker), alcohol intake (nondrinker, current drinker), and history of cancer, CVD, or diabetes (yes, no). Subgroup analyses were performed to examine whether associations varied by sex, age, BMI, smoking status, alcohol intake, and history of cancer, CVD, or diabetes, adjusting for the same potential confounding variables as aforementioned. Tests for heterogeneity by subgroups were evaluated with likelihood-ratio test by comparing the models with and without an interaction term. All statistical analyses were performed using Statistical Analysis System (SAS) version 9.4 (SAS Institute). Statistical significance was defined as a P-value < 0.05 in a two-sided test.

## Results

### Dietary patterns

Factor analysis identified three dietary patterns based on 22 food groups. The first pattern was named as “animal food and condiment pattern,” having high intakes of oils, seasonings, red meat, sugars, and vegetables. The second pattern was highly loaded by intakes of fishes and shellfish, seaweeds, vegetables, fruits, and seasonings and was labeled as “seafood and vegetable pattern.” The last dietary pattern was associated with dairy products, fats, processed meat, fruits, and potatoes and was characterized as “dairy product and processed meat pattern.” Table 1 indicates factor loadings for each food group more than 0.25 or less than − 0.25. These three dietary patterns explained 9.55%, 6.91%, and 5.85% of the variance, respectively.

**Table 1:** Factor-loading matric for the major dietary patterns identified by factor analysis^a^

	Animal food and condiment pattern	Seafood and vegetable pattern	Dairy products and processed meat pattern
Whole grains	.	.	.
Refined grains	.	.	.
Potatoes	.	.	0.31
Sugars	0.45	.	.
Legumes	.	.	.
Nuts and seeds	.	.	.
Vegetables	0.40	0.42	.
Mushrooms	.	.	.
Fruits	.	0.30	0.39
Seaweeds	.	0.66	.
Alcoholic beverages	0.43	.	-0.26
Non-alcoholic beverages	.	.	0.27
Seasonings	0.62	0.27	.
Oils	0.68	.	.
Red meat	0.53	.	.
Processed meat	.	.	0.41
Poultry meat	0.34	.	.
Eggs	0.31	.	0.28
Fishes and shellfish	.	0.69	.
Dairy products	.	.	0.50
Fats	.	.	0.45
Others	.	.	.
**Variance explained (%)**	9.55	6.91	5.85

^a^Absolute values < 0.25 were not listed for simplicity

### Basic characteristics of the study population among quintiles of dietary pattern score

Table 2 shows the sociodemographic and health-related characteristics according to quintiles of each dietary pattern score. In the “animal food and condiment pattern,” participants with higher dietary scores tended to be younger, male, have higher energy intake, higher levels of education and income, and were more likely to be married, smoke, drink, and exercise, and have lower histories of cancer, CVD, or diabetes. Conversely, in the “seafood and vegetable pattern,” individuals with higher dietary scores tended to be older, female, have greater energy intake, higher income levels, higher supplement use, and were more likely to be married and have a higher incidence of cancer, CVD, or diabetes. Lastly, in the “dairy product and processed meat pattern,” those with higher dietary scores tended to be younger, have higher levels of education and income, higher supplement use, and less likely to smoke and drink.

**Table 2:** Demographic and lifestyle characteristics of study participants aged 19 years and older from KNHANES (2007–2015) according to quintiles of dietary pattern score^a^

	Dietary pattern score				
	Quintile 1	Quintile 2	Quintile 3	Quintile 4	Quintile 5
Participants (n)	8924	8925	8925	8925	8924
**Animal food and condiment pattern**					
	← mean ± SE^**b**^ →				
Age (years)	52.0 ± 0.3	49.1 ± 0.2	45.7 ± 0.2	43.0 ± 0.2	39.5 ± 0.2
	← n (weighted %) →				
Sex					
Men	1894 (26.00)	2650 (35.32)	3428 (45.12)	4313 (56.17)	5734 (72.01)
Women	7030 (74.00)	6275 (64.68)	5497 (54.88)	4612 (43.83)	3190 (27.99)
	← mean ± SE^**b**^ →				
BMI^c^ (kg/m^2^)	23.7 ± 0.0	23.5 ± 0.1	23.6 ± 0.0	23.6 ± 0.0	24.0 ± 0.0
Energy intake (kcal/d)	1405.84 ± 9.28	1561.70 ± 7.38	1781.07 ± 7.55	2100.27 ± 8.30	2922.05 ± 12.54
Smoking status (pack-years)	4.7 ± 0.5	5.7 ± 0.4	6.7 ± 0.5	8.2 ± 1.0	9.0 ± 0.3
	← n (weighted %) →				
Education level					
Elementary school or below	3770 (33.74)	3141 (27.01)	2183 (18.10)	1487 (11.39)	875 (6.53)
Middle school	1077 (12.16)	965 (10.69)	1045 (10.81)	930 (9.24)	704 (6.94)
High school	2228 (32.77)	2567 (35.84)	2853 (37.13	3246 (42.60)	3474 (44.36)
College or above	1436 (21.33)	1872 (26.46)	2529 (33.96)	2937 (36.77)	3492 (42.17)
Household income					
Low	2807 (25.57)	2355 (20.59)	1751 (15.77)	1295 (12.21)	849 (8.13)
Low middle	2286 (27.08)	2290 (26.57)	2242 (25.67)	2229 (25.79)	2090 (23.87)
Upper middle	1863 (23.21)	2162 (27.33)	2408 (29.60)	2575 (30.64)	2824 (33.08)
High	1781 (24.14)	1985 (25.51)	2411 (28.96)	2726 (31.36)	3068 (34.92)
Marital status					
Not married	695 (15.03)	842 (16.61)	1044 (19.27)	1362 (23.45)	1853 (29.01)
Married	8200 (84.97)	8051 (83.39)	7858 (80.73)	7532 (76.55)	7046 (70.99)
Current alcohol intake					
None	3948 (39.76)	3280 (32.31)	2506 (24.47)	1951 (18.40)	1234 (11.53)
< 3 drinks/week	3400 (42.43)	3649 (44.07)	3742 (42.89)	3622 (40.73)	3019 (33.32)
3 to <7 drinks/week	563 (8.89)	667 (10.29)	983 (15.04)	1202 (17.36)	1604 (21.12)
≥ 7 drinks/week	673 (8.92)	1020 (13.34)	1434 (17.60)	1893 (23.52)	2788 (34.03)
Vigorous physical activity					
No	6867 (78.67)	6691 (76.01)	6464 (72.92)	6166 (69.39)	5695 (64.91)
Yes	1646 (21.33)	1858 (23.99)	2143 (27.08)	2429 (30.61)	2860 (35.09)
Supplement use					
No	5856 (65.31)	6063 (68.29)	5924 (67.71)	5823 (66.82)	5829 (66.44)
Yes	2953 (34.69)	2778 (31.71)	2895 (32.29)	3018 (33.18)	3015 (33.56)
History of cancer, CVD^d^, or diabetes					
No	3458 (67.88)	3783 (72.88)	3839 (76.41)	3846 (80.74)	3860 (84.65)
Yes	1869 (32.12)	1660 (27.12)	1360 (23.59)	1162 (19.26)	881 (15.35)
**Seafood and vegetable pattern**					
	← mean ± SE →				
Age (years)	40.4 ± 0.2	45.7 ± 0.2	46.3 ± 0.2	46.4 ± 0.2	47.4 ± 0.2
	← n (weighted %) →				
Sex					
Men	1894 (26.00)	2650 (35.32)	3428 (45.12)	4313 (56.17)	5734 (72.01)
Women	7030 (74.00)	6275 (64.68)	5497 (54.88)	4612 (43.83)	3190 (27.99)
	← mean ± SE				
BMI (kg/m^2^)	23.4 ± 0.1	23.6 ± 0.1	23.7 ± 0.0	23.8 ± 0.0	24.0 ± 0.0
Energy intake (kcal/d)	1783.15 ± 13.82	1753.98 ± 11.34	1957.53 ± 10.94	2161.93 ± 11.10	2570.63 ± 13.13
Smoking status (pack-years)	5.6 ± 0.3	7.1 ± 1.1	7.5 ± 0.6	7.1 ± 0.2	8.3 ± 0.3
	← n (weighted %) →				
Education level					
Elementary school or below	2397 (16.91)	2782 (21.87)	2479 (19.50)	2131 (17.14)	1667 (13.57)
Middle school	687 (6.82)	882 (8.97)	1062 (11.21)	1011 (10.71)	1079 (10.98)
High school	3062 (44.39)	2685 (37.76)	2760 (37.91)	2868 (37.37)	2993 (38.13)
College or above	2351 (31.88)	2217 (31.40)	2291 (31.37)	2603 (34.78)	2804 (37.32)
Household income					
Low	2186 (18.42)	2173 (18.94)	1892 (16.01)	1504 (12.61)	1302 (11.03)
Low middle	2316 (28.31)	2265 (25.94)	2304 (26.27)	2198 (24.86)	2054 (22.53)
Upper middle	2228 (28.02)	2224 (27.89)	2359 (29.79)	2503 (30.35)	2518 (30.59)
High	2040 (25.25)	2120 (27.23)	2242 (27.93)	2602 (32.18)	2967 (35.85)
Marital status					
Not married	2072 (36.09)	1167 (21.92)	935 (17.60)	882 (16.73)	740 (13.71)
Married	6826 (63.91)	7732 (78.08)	7959 (82.40)	8014 (83.27)	8156 (86.29)
Current alcohol intake					
None	2428 (20.82)	2780 (25.18)	2669 (24.28)	2592 (24.73)	2450 (23.54)
< 3 drinks/week	3311 (39.65)	3555 (41.82)	3555 (40.54)	3528 (39.92)	3483 (38.68)
3 to <7 drinks/week	1156 (17.66)	906 (14.30)	929 (14.30)	984 (14.58)	1044 (15.33)
≥ 7 drinks/week	1677 (21.86)	1383 (18.69)	1513 (20.87)	1551 (20.76)	1684 (22.45)
Vigorous physical activity					
No	6522 (73.83)	6527 (72.78)	6388 (70.44)	6239 (69.79)	6207 (70.44)
Yes	1973 (26.17)	2045 (27.22)	2204 (29.56)	2374 (30.21)	2340 (29.26)
Supplement use					
No	6403 (72.25)	6138 (68.80)	5943 (67.17)	5709 (64.99)	5302 (60.94)
Yes	2431 (27.75)	2693 (31.20)	2891 (32.83)	3101 (35.02)	3543 (39.06)
History of cancer, CVD, or diabetes					
No	3682 (80.89)	3831 (77.18)	3932 (77.61)	3888 (76.93)	3453 (73.23)
Yes	1179 (19.11)	1400 (22.82)	1431 (22.39)	1404 (23.07)	1518 (26.77)
**Dairy products and processed meat pattern**					
	← mean ± SE →				
Age (years)	48.2 ± 0.3	48.9 ± 0.2	45.7 ± 0.2	43.7 ± 0.2	40.2 ± 0.2
	← n (weighted %) →				
Sex					
Men	4841 (63.40)	3355 (45.91)	3216 (44.76)	3215 (45.25)	3392 (48.88)
Women	4083 (36.60)	5570 (54.09)	5709 (55.24)	5710 (54.75)	5532 (51.12)
	← mean ± SE →				
BMI (kg/m^2^)	24.0 ± 0.0	23.8 ± 0.0	23.7 ± 0.1	23.6 ± 0.0	23.5 ± 0.0
Energy intake (kcal/d)	2161.62 ± 15.97	1713.70 ± 10.79	1849.07 ± 10.41	2006.55 ± 10.47	2417.92 ± 12.89
Smoking status (pack-years)	11.7 ± 0.5	6.9 ± 0.2	7.5 ± 1.1	5.2 ± 0.2	4.7 ± 0.3
	← n (weighted %) →				
Education level					
Elementary school or below	3212 (25.20)	3194 (26.32)	2361 (18.84)	1714 (13.55)	975 (7.10)
Middle school	1092 (12.31)	1018 (11.06)	954 (9.88)	918 (8.94)	739 (6.76)
High school	2561 (37.27)	2545 (36.94)	2810 (37.69)	3092 (39.87)	3360 (43.59)
College or above	1656 (25.22)	1765 (25.68)	2433 (33.59)	2880 (37.64)	3532 (42.54)
Household income					
Low	2540 (20.78)	2418 (20.81)	1836 (16.09)	1322 (12.04)	941 (8.87)
Low middle	2293 (27.00)	2338 (27.45)	2213 (25.13)	2207 (25.06)	2086 (23.84)
Upper middle	2053 (27.24)	2046 (25.79)	2454 (30.54)	2614 (30.96)	2665 (31.39)
High	1868 (24.97)	1974 (25.95)	2323 (28.23)	2667 (31.94)	3139 (35.91)
Marital status					
Not married	898 (17.67)	782 (15.69)	1093 (20.47)	1279 (22.90)	1744 (29.36)
Married	7985 (82.33)	8111 (84.31)	7806 (79.53)	7623 (77.10)	7162 (70.64)
Current alcohol intake					
None	2150 (18.18)	2934 (27.02)	2809 (26.15)	2575 (24.27)	2451 (22.87)
< 3 drinks/week	2382 (26.09)	3348 (38.89)	3625 (42.15)	4002 (45.74)	4075 (46.34)
3 to <7 drinks/week	1061 (15.97)	875 (13.87)	973 (14.97)	1004 (14.96)	1106 (16.47)
≥ 7 drinks/week	3004 (39.76)	1431 (20.21)	1239 (16.73)	1081 (15.04)	1053 (14.32)
Vigorous physical activity					
No	6407 (70.78)	6505 (73.29)	6510 (73.15)	6308 (71.25)	6153 (69.74)
Yes	2118 (29.22)	2019 (26.71)	2050 (26.85)	2296 (28.75)	2451 (30.26)
Supplement use					
No	6802 (75.01)	6315 (70.97)	5848 (66.86)	5394 (63.13)	5136 (59.93)
Yes	2055 (24.99)	2514 (29.03)	2974 (33.14)	3426 (36.87)	3690 (40.07)
History of cancer, CVD, or diabetes					
No	4003 (75.14)	3746 (73.00)	3684 (77.29)	3725 (78.76)	3628 (81.74)
Yes	1596 (24.86)	1689 (27.00)	1357 (22.71)	1264 (21.24)	1026 (18.26)

^a^Continuous variables are given as weighted mean ± SE, and categorical variables are given as weighted percentage. Analysis of variance test was performed to test for group differences across the quintiles of dietary pattern score. Significant differences were detected for all variables (P for all other variables < 0.0001)

^b^SE = standard error

^c^BMI = body mass index; calculated as kg/m^2^

^d^CVD = cardiovascular disease

### Association between dietary patterns and mortality

Table 3 shows the HRs of mortality according to quintiles of the three major dietary patterns. After adjusting for potential confounding factors, the HRs (95% CIs) for all-cause mortality, based on increasing quintiles of the “animal food and condiment pattern,” were 0.98 (0.87–1.10), 0.88 (0.76–1.02), 0.90 (0.76–1.08), and 0.88 (0.70–1.12) (P for trend = 0.208), when compared with the lowest quintile. The “dairy product and processed meat pattern” showed no significant association with all-cause mortality, with multivariate HRs (95% CIs) values of 0.98 (0.86–1.11), 0.91 (0.79–1.04), 0.92 (0.79–1.08), and 0.87 (0.72–1.05) (P for trend = 0.097). Conversely, “seafood and vegetable pattern” illustrated a significant association with all-cause mortality, demonstrating multivariate HRs (95% CIs) values of 0.86 (0.75–0.99), 0.75 (0.65–0.87), 0.81 (0.69–0.94), and 0.87 (0.73–1.05), respectively (P for trend = 0.191).

The multivariate HRs (95% CIs) for CVD mortality according to increasing quintiles of “animal food and condiment pattern” were 0.91 (0.71–1.17), 0.97 (0.71–1.33), 1.06 (0.74–1.53), and 0.82 (0.47–1.43), respectively (P for trend = 0.724), compared with the lowest quintile. The “seafood and vegetable pattern” significantly lowered CVD mortality, showing multivariate HRs (95% CIs) values of 0.82 (0.62–1.07), 0.80 (0.58–1.08), 0.70 (0.50–0.99), and 0.63 (0.42–0.96), respectively (P for trend = 0.027). “Dairy product and processed meat pattern” showed no significant association with CVD mortality, with multivariate HRs (95% CIs) values of 0.81 (0.63–1.04), 0.83 (0.61–1.12), 0.89 (0.64–1.25), and 0.80 (0.52–1.22) (P for trend = 0.348).

In the analysis of cancer mortality, no significant association was observed for any of the three dietary patterns. The multivariate HRs (95% CIs) across ascending quintiles of the “animal food and condiment pattern” were as follows: 1.15 (0.92–1.44), 0.98 (0.75–1.29), 1.02 (0.77–1.37), and 0.93 (0.64–1.36), respectively (P for trend = 0.549), in comparison to the lowest quintile. Additionally, for the “seafood and vegetable pattern,” the multivariate HRs (95% CIs) values were 0.98 (0.74–1.30), 0.86 (0.65–1.14), 0.85 (0.63–1.13), and 1.06 (0.77–1.45) (P for trend = 0.808). The “dairy product and processed meat pattern” demonstrated multivariate HRs (95% CIs) values of 1.04 (0.82–1.31), 1.00 (0.78–1.29), 1.04 (0.79–1.37), and 0.89 (0.63–1.26) (P for trend = 0.587).

**Table 3:** Hazard ratios (95% CIs) for all-cause, cardiovascular disease, and cancer mortality according to the quintile(Q) of dietary pattern scores in aged 19 years or older from the Korea National Health and Nutrition Examination Survey (2007–2015)

	Dietary pattern score					
	Quintile 1	Quintile 2	Quintile 3	Quintile 4	Quintile 5	*P* trend^a^
**All-cause mortality**						
**Animal food and condiment pattern**						
No. of deaths	874	708	484	350	206	
Model 1^b^	1.00 (reference)	0.93 (0.83–1.05)	0.77 (0.67–0.89)	0.74 (0.63–0.86)	0.65 (0.53–0.80)	< 0.001
Model 2^c^	1.00 (reference)	0.98 (0.87–1.10)	0.88 (0.76–1.02)	0.90 (0.76–1.08)	0.88 (0.70–1.12)	0.208
**Seafood and vegetable pattern**						
No. of deaths	686	641	489	440	366	
Model 1^b^	1.00 (reference)	0.79 (0.69–0.90)	0.64 (0.56–0.74)	0.64 (0.55–0.74)	0.64 (0.55–0.74)	< 0.0001
Model 2^c^	1.00 (reference)	0.86 (0.75–0.99)	0.75 (0.65–0.87)	0.81 (0.69–0.94)	0.87 (0.73–1.05)	0.191
**Dairy products and processed meat pattern**						
No. of deaths	869	734	473	336	210	
Model 1^b^	1.00 (reference)	0.95 (0.84–1.08)	0.83 (0.72–0.95)	0.79 (0.68–0.91)	0.69 (0.57–0.82)	< 0.0001
Model 2^c^	1.00 (reference)	0.98 (0.86–1.11)	0.91 (0.79–1.04)	0.92 (0.79–1.08)	0.87 (0.72–1.05)	0.097
**CVD** ^**d**^ **mortality**						
**Animal food and condiment pattern**						
No. of deaths	218	155	116	75	31	
Model 1^b^	1.00 (reference)	0.84 (0.66–1.08)	0.82 (0.61–1.10)	0.83 (0.59–1.16)	0.56 (0.34–0.94)	0.024
Model 2^c^	1.00 (reference)	0.91 (0.71–1.17)	0.97 (0.71–1.33)	1.06 (0.74–1.53)	0.82 (0.47–1.43)	0.724
**Seafood and vegetable pattern**						
No. of deaths	170	155	124	85	61	
Model 1^b^	1.00 (reference)	0.74 (0.57–0.97)	0.68 (0.51–0.91)	0.57 (0.42–0.78)	0.45 (0.31–0.65)	< 0.0001
Model 2^c^	1.00 (reference)	0.82 (0.62–1.07)	0.80 (0.58–1.08)	0.70 (0.50–0.99)	0.63 (0.42–0.96)	0.027
**Dairy product and processed meat pattern**						
No. of deaths	214	167	98	73	43	
Model 1^b^	1.00 (reference)	0.78 (0.61-1.00)	0.74 (0.54-1.00)	0.74 (0.53–1.03)	0.59 (0.39–0.88)	0.008
Model 2^c^	1.00 (reference)	0.81 (0.63–1.04)	0.83 (0.61–1.12)	0.89 (0.64–1.25)	0.80 (0.52–1.22)	0.348
**Cancer mortality**						
**Animal food and condiment pattern**						
No. of deaths	225	226	153	139	84	
Model 1^b^	1.00 (reference)	1.12 (0.90–1.40)	0.92 (0.71–1.20)	0.94 (0.72–1.23)	0.83 (0.60–1.16)	0.148
Model 2^c^	1.00 (reference)	1.15 (0.92–1.44)	0.98 (0.75–1.29)	1.02 (0.77–1.37)	0.93 (0.64–1.36)	0.549
**Seafood and vegetable pattern**						
No. of deaths	166	188	161	157	155	
Model 1^b^	1.00 (reference)	0.93 (0.70–1.23)	0.80 (0.61–1.05)	0.76 (0.57-1.00)	0.89 (0.67–1.18)	0.343
Model 2^c^	1.00 (reference)	0.98 (0.74–1.30)	0.86 (0.65–1.14)	0.85 (0.63–1.13)	1.06 (0.77–1.45)	0.808
**Dairy product and processed meat pattern**						
No. of deaths	244	218	160	125	80	
Model 1^b^	1.00 (reference)	1.01 (0.80–1.26)	0.94 (0.73–1.20)	0.95 (0.73–1.23)	0.79 (0.57–1.09)	0.149
Model 2^c^	1.00 (reference)	1.04 (0.82–1.31)	1.00 (0.78–1.29)	1.04 (0.79–1.37)	0.89 (0.63–1.26)	0.587

^a^P for trend from the first to the highest quintile of dietary pattern score was estimated by treating the median value of each quintile as a continuous variable in the regression model

^b^Model 1: stratified by age at baseline (19 to < 60 years, ≥ 60 years) and further adjusted for age (continuous, years) and sex

^c^Model 2: Model 1 further adjusted for total energy intake (continuous, kcal/d), baseline year (2007, 2008, 2009, 2010, 2011, 2012, 2013, 2014, 2015), body mass index (10 to < 18.5, 18.5 to < 23, 23 to < 25, 25 to < 30, ≥30), smoking status (never, past < 15 pack-years [py], past ≥ 15 py, current < 18 py, current ≥ 18 py for men; never, past < 1.05 py, past ≥ 1.05 py, current < 5.40 py, current ≥ 5.40 py for women), alcohol intake (none, current < 3 drinks/week, 3 to< 7 drinks/week, current ≥ 7 drinks/week), marital status (married, not married), education (elementary school or below, middle school, high school or above), household income (low, lower middle, upper middle, high), vigorous physical activity (yes, no), history of cancer, CVD, or diabetes (yes, no), and the use of supplements (yes, no)^d^CVD = cardiovascular disease

### Association of dietary patterns with all-cause mortality – subgroup analysis

We further investigated potential effect modification by sex, baseline age, BMI, smoking status, alcohol consumption, and history of cancer, CVD, or diabetes on the associations between dietary pattern scores and all-cause mortality (Table 4). In men, the “seafood and vegetable pattern” showed significant results for all-cause mortality. Specifically, the multivariate hazard ratios (HR) (95% CI) were 0.81 (0.66–0.98), 0.74 (0.60–0.91), 0.78 (0.63–0.96), and 0.89 (0.71–1.12), respectively (P for trend = 0.274). A similar trend was observed in women, showing multivariate HRs (95% CIs) values of 0.91 (0.75–1.10), 0.71 (0.57–0.88), 0.81 (0.63–1.04), and 0.78 (0.57–1.06), respectively (P for trend = 0.024). No significant associations were found in other dietary patterns, and notably, no heterogeneity by sex was observed for any of the three dietary patterns. In contrast, significant interactions were observed between all three dietary pattern scores and all-cause mortality in age; with HRs being lower among individuals aged 60 years or older compared to those below 60. Additionally, significant interactions were detected in smoking status, particularly within the “animal food and condiment pattern,” where HRs were lower among nonsmokers than among current smokers. However, no effect modification was observed by BMI, alcohol drinking, and history of chronic disease (i.e., cancer, CVD, or diabetes) in any of the three dietary pattern scores. Although there was no significant interaction with smoking status, a differential effect emerged within the “seafood and vegetable pattern.” No significant associations were found among smokers, whereas nonsmokers consistently showed a significant reduction in all-cause mortality risk.

**Table 4:** Subgroup analysis of all-cause mortality according to the quintile of dietary pattern scores in aged 19 years or older from the Korea National Health and Nutrition Examination Survey (2007–2015)^a^

	Dietary pattern score						
Variable	Quintile 1	Quintile 2	Quintile 3	Quintile 4	Quintile 5	*P* for trend	*P* for interaction
**Animal food and condiment pattern**							
**Sex**							0.667
Men							
Cases, n	342	380	318	255	178		
HR (95% CI)	1.00 (reference)	1.02 (0.86–1.22)	0.87 (0.71–1.06)	0.86 (0.69–1.08)	0.89 (0.67–1.18)	0.127	
Women							
Cases, n	532	328	166	95	28		
HR (95% CI)	1.00 (reference)	0.94 (0.80–1.11)	0.92 (0.73–1.16)	1.03 (0.77–1.38)	0.89 (0.53–1.50)	0.688	
**Age at baseline**							< 0.001
< 60 y							
Cases, n	63	65	71	78	67		
HR (95% CI)	1.00 (reference)	1.11 (0.73–1.71)	0.87 (0.56–1.35)	1.10 (0.70–1.71)	0.99 (0.57–1.72)	0.964	
≥ 60 y							
Cases, n	811	643	413	272	139		
HR (95% CI)	1.00 (reference)	0.97 (0.86–1.10)	0.88 (0.76–1.03)	0.82 (0.67-1.00)	0.87 (0.68–1.11)	0.036	
**BMI** ^b^							0.563
< 25							
Cases, n	643	522	350	236	154		
HR (95% CI)	1.00 (reference)	0.98 (0.85–1.12)	0.88 (0.74–1.04)	0.84 (0.68–1.02)	0.99 (0.76–1.30)	0.203	
≥ 25							
Cases, n	223	180	128	111	50		
HR (95% CI)	1.00 (reference)	0.95 (0.75–1.21)	0.87 (0.66–1.16)	1.03 (0.74–1.43)	0.67 (0.44–1.04)	0.328	
**Smoking status**							0.014
None							
Cases, n	671	522	326	232	112		
HR (95% CI)	1.00 (reference)	0.95 (0.83–1.10)	0.85 (0.71–1.01)	0.81 (0.66-1.00)	0.84 (0.62–1.14)	0.035	
Current							
Cases, n	112	137	128	104	85		
HR (95% CI)	1.00 (reference)	1.21 (0.91–1.61)	1.08 (0.78–1.51)	1.25 (0.86–1.82)	1.10 (0.71–1.70)	0.584	
**Alcohol consumption**							0.598
None							
Cases, n	504	366	209	124	55		
HR (95% CI)	1.00 (reference)	1.02 (0.87–1.20)	1.12 (0.91–1.38)	0.96 (0.74–1.26)	1.23 (0.84–1.80)	0.466	
Current							
Cases, n	330	303	255	219	144		
HR (95% CI)	1.00 (reference)	0.90 (0.75–1.09)	0.71 (0.58–0.88)	0.83 (0.65–1.05)	0.69 (0.50–0.95)	0.014	
**History of cancer, CVD, or diabetes** ^c^							0.733
No							
Cases, n	280	248	182	149	83		
HR (95% CI)	1.00 (reference)	1.00 (0.80–1.23)	0.98 (0.77–1.26)	1.10 (0.83–1.47)	0.89 (0.60–1.31)	0.999	
Yes							
Cases, n	184	166	115	68	42		
HR (95% CI)	1.00 (reference)	1.06 (0.81–1.39)	0.90 (0.67–1.22)	0.79 (0.55–1.13)	0.88 (0.54–1.43)	0.205	
**Seafood and vegetable pattern**							
**Sex**							0.577
Men							
Cases, n	314	338	282	283	256		
HR (95% CI)	1.00 (reference)	0.81 (0.66–0.98)	0.74 (0.60–0.91)	0.78 (0.63–0.96)	0.89 (0.71–1.12)	0.274	
Women							
Cases, n	372	303	207	157	110		
HR (95% CI)	1.00 (reference)	0.91 (0.75–1.10)	0.71 (0.57–0.88)	0.81 (0.63–1.04)	0.78 (0.57–1.06)	0.024	
**Age at baseline**							< 0.001
< 60 y							
Cases, n	73	69	67	66	69		
HR (95% CI)	1.00 (reference)	0.91 (0.61–1.37)	0.83 (0.54–1.26)	0.89 (0.58–1.36)	1.07 (0.68–1.69)	0.818	
≥ 60 y							
Cases, n	613	572	413	272	139		
HR (95% CI)	1.00 (reference)	0.86 (0.75-1.00)	0.72 (0.62–0.84)	0.79 (0.66–0.93)	0.83 (0.69–1.01)	0.008	
**BMI** ^b^							0.179
< 25							
Cases, n	498	502	353	303	249		
HR (95% CI)	1.00 (reference)	0.93 (0.79–1.10)	0.76 (0.64–0.92)	0.79 (0.65–0.96)	0.88 (0.71–1.10)	0.042	
≥ 25							
Cases, n	179	134	131	131	117		
HR (95% CI)	1.00 (reference)	0.69 (0.53–0.91)	0.67 (0.51–0.89)	0.80 (0.61–1.06)	0.85 (0.61–1.16)	0.412	
**Smoking status**							0.298
None							
Cases, n	454	470	350	322	267		
HR (95% CI)	1.00 (reference)	0.81 (0.69–0.95)	0.64 (0.54–0.76)	0.73 (0.60–0.88)	0.77 (0.62–0.96)	0.004	
Current							
Cases, n	169	123	113	87	84		
HR (95% CI)	1.00 (reference)	0.93 (0.70–1.24)	1.05 (0.77–1.43)	0.86 (0.63–1.19)	1.16 (0.81–1.66)	0.699	
**Alcohol consumption**							0.193
None							
Cases, n	370	339	220	202	127		
HR (95% CI)	1.00 (reference)	0.88 (0.73–1.06)	0.65 (0.53–0.79)	0.75 (0.60–0.94)	0.74 (0.56–0.98)	0.003	
Current							
Cases, n	280	274	253	215	229		
HR (95% CI)	1.00 (reference)	0.83 (0.67–1.02)	0.82 (0.66–1.02)	0.80 (0.63-1.00)	0.97 (0.76–1.24)	0.652	
**History of cancer**,** CVD**,** or diabetes**^c^							0.122
No							
Cases, n	243	239	184	167	109		
HR (95% CI)	1.00 (reference)	0.95 (0.76–1.18)	0.86 (0.67–1.10)	0.87 (0.68–1.11)	0.78 (0.57–1.08)	0.107	
Yes							
Cases, n	49	50	45	46	54		
HR (95% CI)	1.00 (reference)	0.66 (0.48–0.91)	0.53 (0.39–0.73)	0.66 (0.47–0.92)	0.85 (0.60–1.20)	0.218	
**Dairy products and processed meat pattern**							
**Sex**							0.714
Men							
Cases, n	538	358	266	190	121		
HR (95% CI)	1.00 (reference)	0.86 (0.72–1.03)	0.97 (0.81–1.16)	0.99 (0.80–1.21)	0.89 (0.69–1.15)	0.645	
Women							
Cases, n	331	376	207	146	89		
HR (95% CI)	1.00 (reference)	1.09 (0.91–1.31)	0.82 (0.66–1.01)	0.80 (0.63–1.03)	0.81 (0.60–1.10)	0.014	
**Age at baseline**							< 0.001
< 60 years							
Cases, n	95	86	60	66	37		
HR (95% CI)	1.00 (reference)	1.26 (0.89–1.80)	0.87 (0.58–1.29)	1.05 (0.71–1.55)	0.75 (0.47–1.20)	0.209	
≥ 60 years							
Cases, n	774	648	413	270	173		
HR (95% CI)	1.00 (reference)	0.92 (0.80–1.05)	0.92 (0.80–1.05)	0.87 (0.74–1.03)	0.97 (0.79–1.18)	0.234	
**BMI** ^b^							0.768
< 25							
Cases, n	659	527	338	236	145		
HR (95% CI)	1.00 (reference)	0.96 (0.82–1.12)	0.88 (0.75–1.03)	0.92 (0.76–1.12)	0.85 (0.67–1.07)	0.107	
≥ 25							
Cases, n	201	200	130	98	63		
HR (95% CI)	1.00 (reference)	0.98 (0.78–1.23)	0.94 (0.72–1.22)	0.85 (0.63–1.14)	0.88 (0.61–1.28)	0.287	
**Smoking status**							0.582
None							
Cases, n	454	470	350	322	267		
HR (95% CI)	1.00 (reference)	1.04 (0.89–1.20)	0.94 (0.80–1.10)	0.91 (0.75–1.09)	0.98 (0.80–1.21)	0.339	
Current							
Cases, n	169	123	113	87	84		
HR (95% CI)	1.00 (reference)	0.83 (0.63–1.08)	0.81 (0.60–1.08)	1.00 (0.70–1.42)	0.72 (0.43–1.21)	0.279	
**Alcohol consumption**							0.830
None							
Cases, n	376	382	232	165	103		
HR (95% CI)	1.00 (reference)	0.94 (0.80–1.12)	0.96 (0.79–1.16)	0.92 (0.78–1.16)	0.89 (0.68–1.17)	0.397	
Current							
Cases, n	460	315	220	152	104		
HR (95% CI)	1.00 (reference)	1.00 (0.83–1.21)	0.85 (0.69–1.04)	0.90 (0.71–1.14)	0.93 (0.71–1.22)	0.253	
**History of cancer**,** CVD**,** or diabetes**^c^							0.680
No							
Cases, n	362	241	168	103	68		
HR (95% CI)	1.00 (reference)	0.99 (0.81–1.21)	0.97 (0.78–1.22)	0.88 (0.67–1.15)	0.96 (0.69–1.35)	0.329	
Yes							
Cases, n	179	183	81	77	55		
HR (95% CI)	1.00 (reference)	1.10 (0.84–1.45)	0.71 (0.51–0.97)	1.00 (0.71–1.39)	0.79 (0.55–1.14)	0.093	

^a^Regression models were stratified by age at baseline (19 to < 60 years, ≥ 60 years) and further adjusted for age (continuous, years), total energy intake (continuous, kcal/d), baseline year (2007, 2008, 2009, 2010, 2011, 2012, 2013, 2014, 2015), BMI (10 to < 25, 25 to < 30, ≥30), smoking status (never, past, current), alcohol intake (none, current < 3 drinks/week, 3 to <7 drinks/week, current ≥ 7 drinks/week), marital status (married, not married), education (elementary school or below, middle school, high school or above), household income (low, lower middle, upper middle, high), vigorous physical activity (yes, no), history of cancer, CVD, or diabetes (yes, no), and the use of supplements (yes, no)

^b^BMI = body mass index; calculated as kg/m^2 c^Not adjusted for history of cancer, CVD, or diabetes

## Discussion

In this large prospective study among Korean men and women, “animal food and condiment pattern” and “dairy products and processed meat pattern” did not show significant association with all-cause and CVD mortality. On the other hand, the “seafood and vegetable pattern,” characterized by high intake of fishes and shellfish, seaweeds, vegetables, fruits, and seasonings, was found to be significantly related to a lower risk of all-cause and CVD mortality. Compared with the first quintile of “seafood and vegetable pattern score,” the risk of all-cause mortality decreased with an downward trend across quintiles, with reductions of 14% in the second quintile, 25% in the third quintile, and 19% in the fourth quintile. Likewise, for CVD mortality, the risk decreased by 30% in the fourth quintile and 37% lower in the highest quintile compared to the first quintile of the “seafood and vegetable pattern score.” No significant association was found for any of the three dietary patterns in the cancer mortality analysis. Furthermore, the inverse association between “seafood and vegetable pattern” and all-cause mortality generally did not vary by subgroups, including sex, BMI, smoking status, alcohol consumption, and history of chronic disease, except for age at baseline.

These findings are consistent with previous studies indicating that increased intake of fish, vegetables, and fruits was associated with a decreased risk of all-cause mortality and CVD mortality [23, 24]. Further support for these results is provided by prior research [25] suggesting an inverse association between fish consumption and the risk of all-cause and CVD mortality, particularly among Asian populations [26]. Additionally, intakes of fruits and vegetables were significantly linked to a reduced risk of all-cause and CVD mortality, but not cancer [27]. These findings align with the well-established fact that seafood and vegetable consumption is beneficial for health and can help prevent CVD. Nonetheless, some studies have suggested that fruit and vegetable consumption was associated with a reduced risk of cancer as well as all-cause and CVD mortality [28].

In contrast to the findings of this study, many studies have reported a significant association between red and processed meat consumption and overall mortality as well as mortality from various diseases. However, such differences could stem from the diversity in dietary habits, meat consumption by country, cultural and genetic factors. Although high consumption of red meat and processed meat are known to be associated with an increased risk of all-cause mortality [23, 29–31], no significant associations were observed between other two dietary patterns and all-cause, CVD, or cancer mortality. According to the Food and Agriculture Organization of the United Nations [32], Koreans consume 70.71 kg of meat per capita, while Americans consume 124.11 kg of meat per capita. Moreover, animal food and condiment pattern is more linked to plant oils and seasoning than red meat, poultry meat, and eggs, showing higher factor loading that is seen in our study.

In the subgroup analysis by sex, we found no significant heterogeneity between men and women. The relationship of the seafood and vegetable pattern with all-cause mortality remained consistent whether we analyzed the data combined or separately by gender. Similar results were observed in JPHC study [10], a prospective study of Danish men and women [33], and the Nurses’ Health Study [9]. The “seafood and vegetable pattern” in our study resembled the “prudent” pattern observed in other studies, while the “animal food and condiment pattern” reflected the “Western” pattern in those studies. In the Danish World Health Organization-MONICA survey study, dietary patterns and their associations with mortality were remarkably consistent across sex and age. The JPHC study also demonstrated consistency in CVD mortality between men and women. However, differences have been noted in other studies. The JPHC study showed significant interactions by sex for the Westernized dietary pattern and all-cause mortality. While our study did not exhibit significant results for both men and women, in the JPHC study, only men showed an inverse association with all-cause mortality. The Danish study also demonstrated similar results, indicating that the prudent dietary pattern lowered the risk of CVD in both men and women, while the western dietary pattern was not associated all-cause mortality. In the Nurses’ Health Study, the prudent pattern showed an inverse association with total mortality, and similarly, the Western pattern was also inversely associated with total mortality. However, in the Shanghai Women’s Health Study [34], the “vegetable-rich” diet was not associated with total death. The difference in mortality rates between men and women depends not only on dietary pattern but also significantly on factors such as public health infrastructure and healthcare resources [35]. Since our study could not adjust for these variables, it is possible that these unmeasured factors contributed to the differing results compared to previous studies.

In addition, our results of subgroup analyses underline that seafood and vegetable pattern may lower the risk of morality among the older, especially above 60 years old. These results are in line with those from previous cohort studies [36–38] or meta-analysis [39] reporting the effect of an mediterranean diet or other vegetable-based dietary patterns in reducing mortality risk in the elderly. In the subgroup analysis according to smoking status, non-smokers showed a significant reduction in the relationship between all-cause mortality and the seafood and vegetable pattern, despite no significant interaction with smoking status. Considering findings from a previous study [40] suggesting that higher intake of fruits and vegetables precedes smoking cessation in current smokers, caution is warranted when interpreting these results. Unlike the results of our subgroup analysis, fruits and vegetable intake was inversely associated with total [41] and CVD [41, 42] mortality among smokers in Europe and US cohort studies. Furthermore, evidence from Takayama Study [43] in Japan suggested that vegetable intake might be more protective against CVD mortality in men who had never smoked, although the interaction was not significant. Due to the limited case numbers in this study, a subgroup analysis for CVD and cancer mortality was not conducted. Our study, along with previous research, highlights the need to explore the associations of dietary patterns with CVD and cancer mortality across different subgroups.

Although our subgroup analysis did not reveal significant associations between dietary patterns and mortality by alcohol consumption, alcohol consumption is a critical factor influencing mortality risk. Estimates of mortality risk from alcohol consumption can vary significantly depending on study design and characteristics. A previous meta-analysis of 107 studies found no significant protective associations of occasional or moderate alcochol consumption on all-cause mortalit. It also found an increased risk of all-cause mortality for individuals consuming 45 g or more alcohol per day. Especially, female drinkers had a significantly higher risk of mortality compared to lifetime nondrinkers [44]. Another meta-analysis have demonstrated that low-volume drinkers offer no overall mortality benefit compared to either occasional or former drinkers [45].

### Strengths and limitations

The major strength of this study was utilizing the data from a nationally representative sample of Korean adults, enhancing the generalizability of the findings. The large sample size of 44,623 participants with 2,622 deaths might provide sufficient statistical power. Second, it is a first prospective cohort study to examine the association between dietary patterns and all-cause, CVD, and cancer mortality using the KNHANES data. As a cohort study, it may be less susceptible to selection bias and recall bias compared to case-control design. Third, considering dietary patterns instead of single nutrients or foods appears to have advantages [5]. As nutrient-based recommendations continue to prevail, developing dietary guidelines that focus on foods can help prevent unintended increases in the intake of specific nutrients. By shifting the focus towards a food-centric approach, inadvertent isolated increments in particular nutrients can be mitigated. Finally, the potential impact of undiagnosed diseases on participants’ diets was mitigated by excluding individuals who died within two years of the baseline.

This study has several limitations. First, since dietary intake was evaluated only once at baseline, long-term dietary habits or changes in diet during the follow-up would not have been reflected in this study. Second of all, using factor analysis needs subjective decisions regarding the method of rotation and initial factor extraction [46]. Thirdly, dietary data were obtained through 24-hour recalls, which may not represent usual dietary intake. Although we adjusted for several potential confounders in the multivariable model, we cannot disregard the possibility of residual confoundings, unknown or unmeasured confounders, thus interpretation should be made with caution.

## Conclusions

In this nationally representative cohort, adherence to the “seafood and vegetable” dietary pattern was significantly associated with a reduced risk of all-cause and CVD mortality. Our findings indicate that high consumption of fish, seaweed, vegetables, and fruits may contribute to a lower risk of all-cause and CVD mortality in the Korean population. Further research is required to explore the association between dietary patterns and cause-specific mortality.

## Data Availability

The KNHANES database is publicly available at the KNHANES website (https://knhanes.kdca.go.kr/knhanes/main.do).

## Abbreviations

BMI: Body mass index
CI: Confidence interval
CVD: Cardiovascular disease
HR: Hazard ratio
KNHANES: Korea National Health and Nutrition Examination Survey
NHANES: National Health and Nutrition Examination Survey
NHS: Nurses’ Health Study
SE: Standard error
